# Genetic Screening of Inpatients With Intellectual Disabilities: A Service Development Project to Widen Access to Up-to-Date Genetic Screening in Adults With an Intellectual Disability

**DOI:** 10.1192/bjo.2024.398

**Published:** 2024-08-01

**Authors:** Shauna Monaghan

**Affiliations:** NIMDTA, Belfast, United Kingdom

## Abstract

**Aims:**

Problem:

Around 10% of patients with an intellectual disability have a clinically relevant copy number variant in their DNA detected using microarray analysis. Adults with an intellectual disability may not have had access to genetic screening during their patient journey, or they may have had previous screening with now outdated technology.

Aim: to offer up-to-date genetic screening to adults with intellectual disability in an inpatient setting.

**Methods:**

Strategy for change:

In collaboration with the clinical genetics department, confirm local capacity for genetic screening by microarray testing, and create a pathway for referral with a screening tool for detection of “high risk” patients. Develop processes and resources for consenting patients with capacity, and for acting in best interests with family agreement for those without.

Measurement of Improvement:

Increase in the number of patients with up-to-date genetic screening.

**Results:**

Effects of change:

This project has detected previously unknown genetic abnormalities in current inpatients, two of which were felt to be clinically significant. Further testing is underway and clinical discussions are ongoing regarding the implication of these findings for current patient care and management.

**Conclusion:**

Discussion:

Patients with capacity, families and multidisciplinary healthcare professionals were overwhelmingly supportive of this project with the aim of improving understanding and ultimately the care of each individual. With further discussion and assistance, this project could be widened to the community setting to benefit a greater number of patients and their families.

